# The effects of physicochemical variables and tadpole assemblages on microalgal communities in freshwater temporary ponds through an experimental approach

**DOI:** 10.1186/s12999-014-0013-4

**Published:** 2015-01-20

**Authors:** Bilassé Zongo, Joseph I Boussim

**Affiliations:** Laboratoire de Biologie et Écologie Végétales, Université de Ouagadougou, Ouagadougou, Burkina Faso

**Keywords:** Microalgae, Phytoplankton, Periphyton, Physicochemical variables, Tadpoles assemblages, Experimental approach

## Abstract

**Background:**

In freshwater systems, microalgae are the major biomass of microorganisms. They occur in ecosystems that are largely structured by the climatic regime, the physical and chemical environments with which they interact, and the biological interactions that occur within them. Amphibian larvae are most present in standing water habitats where they are important primary and secondary consumers and even predators. Studies conducted in America and Europe have shown that tadpoles play an important role in the regulation of the algal community structure and water quality in ecosystems. This article aimed to study the effects of the physicochemical variables and tadpole assemblages of four species on microalgae in artificial freshwater ponds using an experimental approach in the Pendjari area, a flora and fauna reserve located in the extreme north-west of Benin.

**Results:**

The species of phytoplankton and periphyton recorded in ponds were among the taxonomical groups of chlorophytes, cyanophytes, euglenophytes, diatoms and dinoflagellates. Chlorophytes were the dominant group in the algal communities. Physicochemical variables affected the biomass of the different communities of algae in temporary freshwater ponds. Transparency and pond size were the most determinative variables of the structure of microalgae communities in ponds. Tadpoles of *Kassina fusca*, *Ptychadena. bibroni*, and *Phrynomantis microps* were important for the regulation of the water quality and algal community structure by grazing and filter-feeding.

**Conclusions:**

A decrease in the tadpole population in the artificial temporary ponds due to predation by carnivorous tadpoles of *Hoplobatrachus occipitalis* caused a disturbance of the algal community structure. This means that the decline of the amphibian population will critically lead to the impoverishment of ecosystems, thereby negatively influencing aquatic and terrestrial ecosystems.

## Background

Microalgae represent an important component in all-aquatic ecosystems, from oceans to small ephemeral ponds. They play a basic role in the functioning of aquatic ecosystems. Within these ecosystems, factors controlling the composition of species types, distribution and biomass of microalgae are physicochemical and biological parameters as well as the hydrologic cycle [1-5]. Microalgae occur in ecosystems that are largely structured by the climatic regime, the physical (e.g., light, temperature, mixing, flow, and habitat) and chemical (e.g., organic and inorganic carbon, oxygen, nutrients) parameters with which they interact and the biological interactions (e.g., grazing and predation) that occur within them [6]. In freshwater systems, microalgae are the major biomass of microorganisms. They are primary producers and an essential part of the diet of primary consumers in aquatic ecosystems [7,8]. Hence, their ecological role is in the balancing of aquatic habitats.

Amphibians are an important and diverse component of both terrestrial and aquatic ecosystems [9]. Amphibian larvae are mostly present in standing freshwater habitats [10] where they are important primary and secondary consumers and even predators [11,12]. The abundance and diversity of microalgae and other primary consumers (e.g., zooplankton, juvenile fishes) are affected by tadpoles [10,13,14].

Studies (e.g., [9,13,15]) on the interactions between tadpoles and algae communities, especially periphyton, have been conducted. Some investigations have shown that grazing amphibian larvae can influence the abundance and community composition of periphyton [10,16]. Some studies have demonstrated that most anurans are filter- feeders [17], playing an important ecological role in the maintenance of water quality. Therefore, catastrophic amphibian losses could have significant effects on water quality and algal assemblage structures [9,11,12,16]. Hence, many studies conducted on the global decline of amphibians (e.g., [9,11,12,18]) have demonstrated the critical consequences on ecosystem properties. To understand the significance of these losses and their actual consequences, more quantitative and qualitative information on the ecological roles of amphibians is urgently needed [11,12].

In tropical savannah regions, temporary ponds are numerous and important to the local population and their cattle. During rainy seasons, particularly in rural areas, pond water is generally used by people for house chores, drinking, bathing (especially by children) and washing. The use of pond water by people and cattle is relatively high for rural field work and domestic animal pastures. Temporary ponds simultaneously constitute major reproduction sites for savannah amphibians. Despite their importance, little research has been conducted on the ecology and functions of tropical ponds [12]. Information about microalgae in these ponds, especially regarding their relationship to physicochemical variables and tadpole assemblages, is particularly unavailable in the Sahelo-Soudanian region of West Africa.

This study aimed to investigate the algal communities of periphyton and phytoplankton and their potential correlation with physicochemical variables and tadpole assemblages in temporary ponds. An experiment was then conducted to better understand the different interactions in temporary ponds.

## Results

### Effects of species exclusion on tadpoles’ survival rate in assemblages

**Figure 1:**
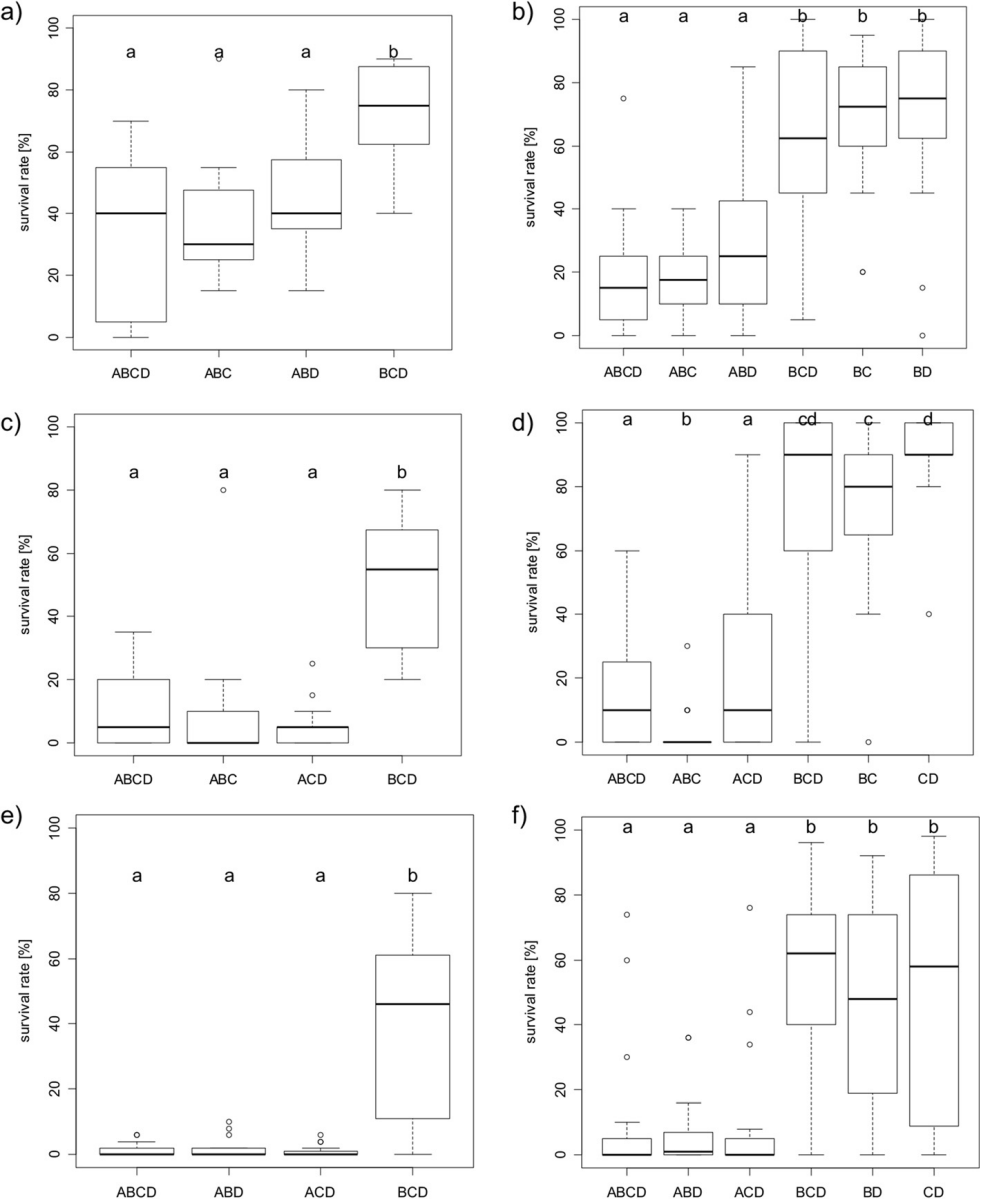
**Comparisons of the survival rate of the respective tadpole species in treatments of varying species compositions [** 19 **].** A: *H. occipitalis*, B: *K. fusca*, C: *P. bibroni*, D: *P. microp*; **a)** Survival rate of *K. fusca* in large tanks, **b)** *K. fusca* in small tanks), **c)** *P. bibroni* in large tanks, **d)** *P. bibroni* in small tanks, **e)** *P. microps* in large tanks (N = 59), **f)** *P. microps* in small tanks. Small letters indicate significant differences between treatments.

According to the results of Mohneke [19], no effects of species composition on the survival rate of the carnivorous *Hoplobatrachus occipitalis* tadpoles were recorded for either large tank (X^2^ test, X^2^ = 0.851, df = 3, p = 0.837) or small tank treatments (X^2^ test, X^2^ = 0.246, df = 3, p = 0.970). Thus, the presence or absence of a particular prey species had no influence on the survival of the predator. An average of 75.06% (±5.98%) and 81.25% (±2.5%) of *H. occipitalis* tadpoles survived in large and small tanks, respectively. The survival rate of *Kassina fusca* differed significantly between treatments of varying species compositions in large tanks (Kruskal-Wallis test, X^2^ = 21.897, df = 3, p < 0.001). Significantly more larvae survived when the predatory tadpole was absent (BCD: median = 75%; BCD vs. ABCD: p < 0.001, BCD vs. ABC: p < 0.001, BCD vs. ABD: p < 0.01, Figure 1a). Among small tank treatments, the survival rate of *K. fusca* likewise differed between treatments of varying species compositions (Kruskal-Wallis test, X^2^ = 64.781, df = 5, p < 0.001). In all predator free communities (BCD: median = 62.5%, BC: median = 72.5%, BD: median = 75%), significantly more tadpoles survived than in the ABCD, ABC and ABD trials. No effects of *P. bibroni* or *P. microps* could be detected in large tanks and small tanks (Figure 1b).

The survival rate of *Ptychadena bibroni* also differed significantly between treatments of varying species compositions in large tanks (Kruskal-Wallis test, X^2^ = 29.158, df = 3, p < 0.001, n = 60). Their survival rate was highest in BCD (median = 55%) and lowest (median = 0%) in ABC (Figure 1c). There were no significant differences in survival between treatments when *H. occipitalis* was present. In small tanks, the survival rate of *P. bibroni* differed significantly between treatments (Kruskal-Wallis, X^2^ = 76.803, df = 5, p < 0.001). In all three predator-free communities (BCD, BC, CD), significantly more tadpoles of *P. bibroni* survived than in all three tadpole assemblages with *H. occipitalis* (ABCD, ABC, ACD) (Figure 1d). The survival rate was significantly lowest, with a median of zero, in the small tank treatment in which *P. microps* was excluded (ABC). Almost all of the tadpoles tested died during the experiment in this community. In BC, the median *P. bibroni* survival was significantly lower than in CD (p < 0.01). The survival rates of *P. microps* tadpoles differed significantly between treatments in large tanks (Kruskal-Wallis, X^2^ = 26.076, df = 3, p < 0.001) as well as in small tanks (Kruskal-Wallis test, X^2^ = 53.684, df = 5, p < 0.001). In general, significantly more tadpoles survived when the larvae of *H. occipitalis* were absent (Figure 1e & f). In almost all tank communities with the predator, the median survival of *P. microps* was zero.

### Microalgae in artificial temporary ponds

All recorded species of phytoplankton and periphyton in artificial temporary ponds were classified into different taxonomic groups. Table 1 shows the distribution and relative abundances of different algal communities recorded in large and small temporary artificial ponds at the end of the experiments. Chlorophytes were the most dominant group of phytoplankton and periphyton. They were predominantly represented by *Mougeotia* sp., *Paulschulzia* sp., *Cosmarium staurastroides*, *Cosmarium quadrum* and *Cosmarium margaritatum*. This group was followed by cyanophytes, which were dominated by *Oscillatoria terebiformis* and *Chroococcus limneticus*. The dominant species of the two groups had high abundances, and their density reached an average of 250 individuals per cm^2^ for periphyton and 385 individuals per ml for phytoplankton. Euglenophytes, diatoms and dinophytes were weakly represented in ponds. Moreover, considering the global richness of microalgae in artificial temporary ponds, the species diversity of experimental groups was comparatively lower than the richness of algae in natural temporary ponds located in the Pendjari area as well as in other savannah areas (e.g., the savannah areas of Burkina Faso where densities of phytoplankton communities are generally too high and can reach some thousands of individuals per millilitre). Nevertheless, the global densities of both phytoplankton and periphyton in ponds increased from the beginning to the end of the experiments (Table 2).

**Table 1:** **Index showing the distribution and relative abundances (n) of algae groups in small and large artificial ponds**

**Types of classification**	**Large artificial ponds**		**Small artificial ponds**	
**Taxonomic groups**	**Phytoplankton**	**Periphyton**	**Phytoplankton**	**Periphyton**
**Cyanophytes**	+++	++	+++	_+++_
**Cyanophytes**		+	+	+++
**Euglenophytes**		+	+	+
**Diatoms**		+	+	+
**Dinophytes**	+	+	+	+

+: n < 100 individuals.ml^−1^ for phytoplankton and n < 25 individuals.cm-^2^ for periphyton; ++: n = 100-200 individuals.ml^−1^ for phytoplankton and n = 25-50 individuals.cm-^2^ for periphyton; +++: n > 200 individuals.ml^−1^ for phytoplankton and n > 50 individuals.cm-^2^ for periphyton; empty cells: no individuals, no species.

**Table 2:** **Mean abundance of algae in artificial ponds at the beginning and end of the experiment**

**Periods**	**Phytoplancton (individuals.ml** ^**−1**^ **)**	**Periphyton (individuals.cm** ^**-2**^ **)**
**Beginning**	523	0
**End (After 14 days)**	938	50

### Physicochemical variables and microalgae in artificial ponds

Table 3 shows the mean values of the physicochemical variables in small and large artificial freshwater ponds in relation to tadpole assemblages.

**Table 3:** **Mean values of environmental variables in small and large artificial ponds following tadpole assemblages**

**Ponds**	**Depth (cm)**	**pH**	**Ec (μS.cm** ^**−1**^ **)**	**Visibility (cm)**	**NO**_**3**_^**−**^ **(mg.L** ^**−1**^ **)**	**PO**_**4-P**_ **(mg.L** ^**−1**^ **)**	**NH**_**4**_^**+**^ **(mg.L** ^**−1**^ **)**
Small artificial ponds (n = 75)							
ABCD	29.92	7.09	13.78	12.08	0.97	0.18	_0.05_
ABC	29.87	7.09	10.06	14.75	0.44	0.25	_0.12_
ABD	29.28	7.08	12.30	14.46	0.64	0.14	_0.08_
ACD	29.57	7.17	11.26	12.94	0.56	0.20	_0.11_
BCD	29.57	7.11	12.07	20.74	0.67	0.18	_0.15_
BC	29.66	7.29	5.62	15.38	1.19	0.40	_0.07_
BD	29.37	7.33	5.67	21.25	0.88	0.18	_0.11_
CD	30.11	7.32	5.53	18.68	0.79	0.33	_0.07_
sd	0.10	0.04	1.21	1.24	0.08	0.03	_0.01_
Large artificial (n = 75)							
ABCD	41.83	7.53	8.29	12.07	1.46	0.15	_0.06_
ABC	37.73	7.23	9.31	8.06	2.03	0.24	_0.08_
ABD	46.31	7.12	2.80	8.00	0.87	0.11	_0.03_
ACD	38.99	7.16	8.60	6.47	2.33	1.82	_0.05_
BCD	39.10	7.39	7.53	11.33	2.80	0.60	_0.06_
sd	1.53	0.08	1.16	1,07	0.34	0.32	0.01

EC: Conductivity; A: *Hoplobatrachus occipitalis*; B: *Kassina fusca*; C: *Ptychadena bibroni*; D: *Phrynomantis microps*. n: sample size; sd: standard deviation.

**Figure 2:**
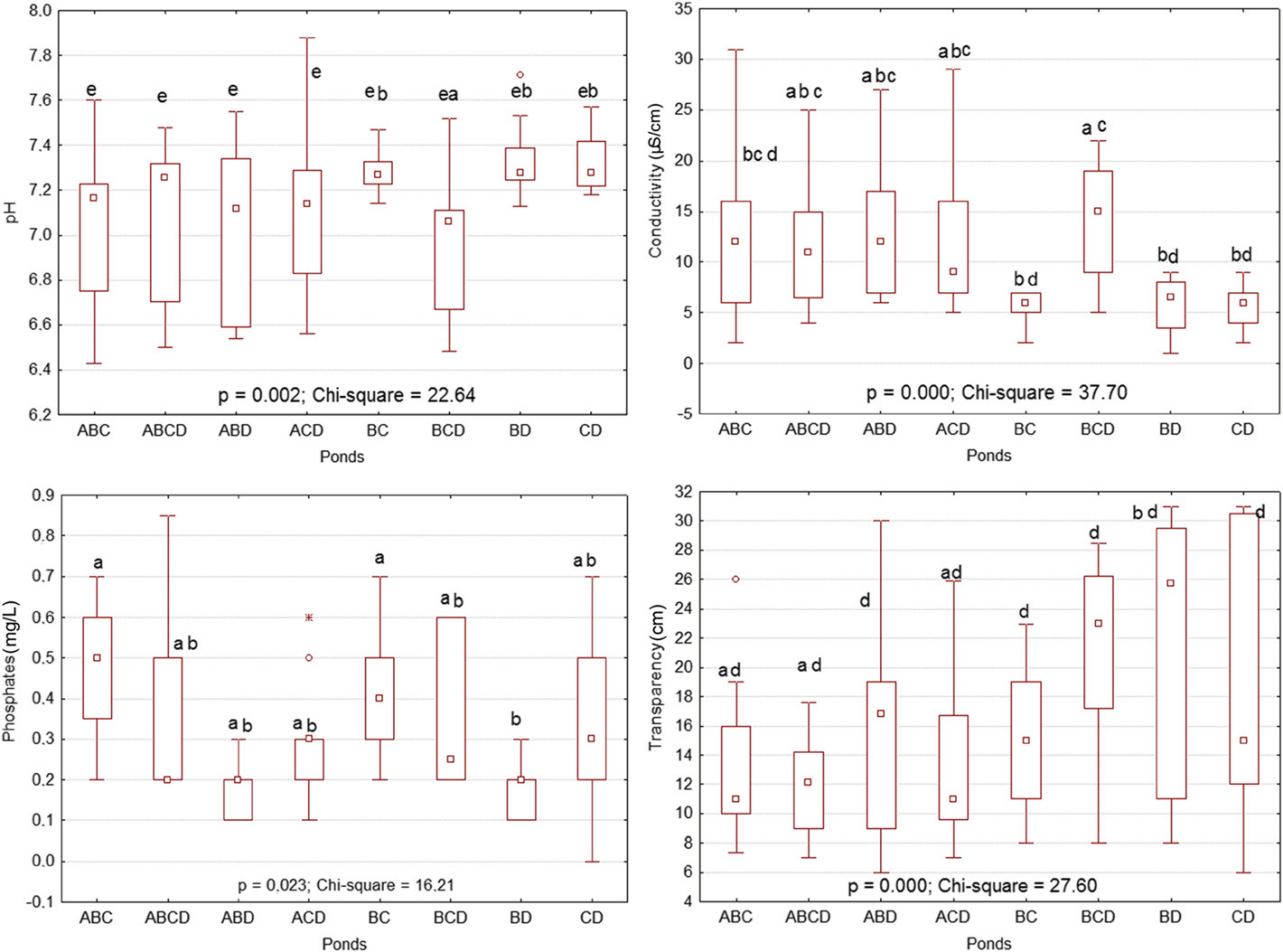
**Differences and similarities of variables between tadpole assemblages in small ponds after comparison using a t-test.** Small letters indicate significant differences between treatments.

**Figure 3:**
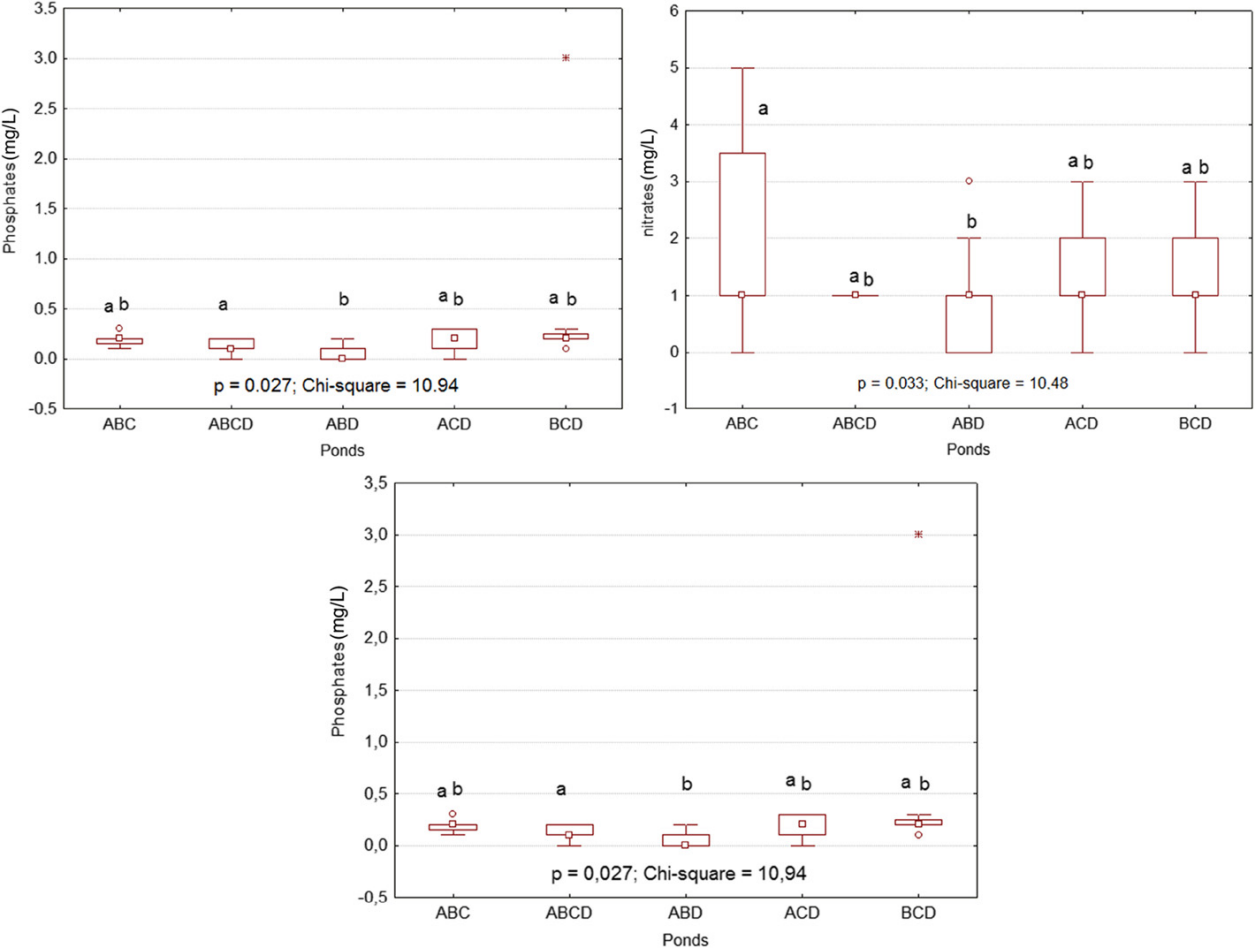
**Differences and similarities of variables between tadpole assemblages in large ponds after comparison using a t-test.** Small letters indicate significant differences between treatments.

In small artificial ponds, the depth ranged from 29.4 to 30 cm, while in the large ones, it ranged from 37.7 to 46.3 cm. According to the tadpole assemblages, the highest mean values of pH, transparency, and concentration of nitrates (NO_3_^−^) and phosphates-phosphorus (PO_4_-P) were noticed in artificial ponds containing BCD, BC, BD, and CD. The highest values of the latest variables were more remarkable in small artificial ponds. Conductivity was higher in ponds containing ABC, ABCD, ABD, and ACD assemblages and was lower in artificial ponds with BCD, BC, BD, and CD assemblages. However, statistical analyses (t-test) indicated that the pH, conductivity, concentration of phosphates-phosphorus and transparency of water significantly differed between tadpole assemblages in small temporary artificial ponds (Figure 2). In large artificial ponds, conductivity and the concentrations of nitrates and phosphates-phosphorus showed significant differences between tadpole assemblages (Figure 3) according to the same test.

**Figure 4:**
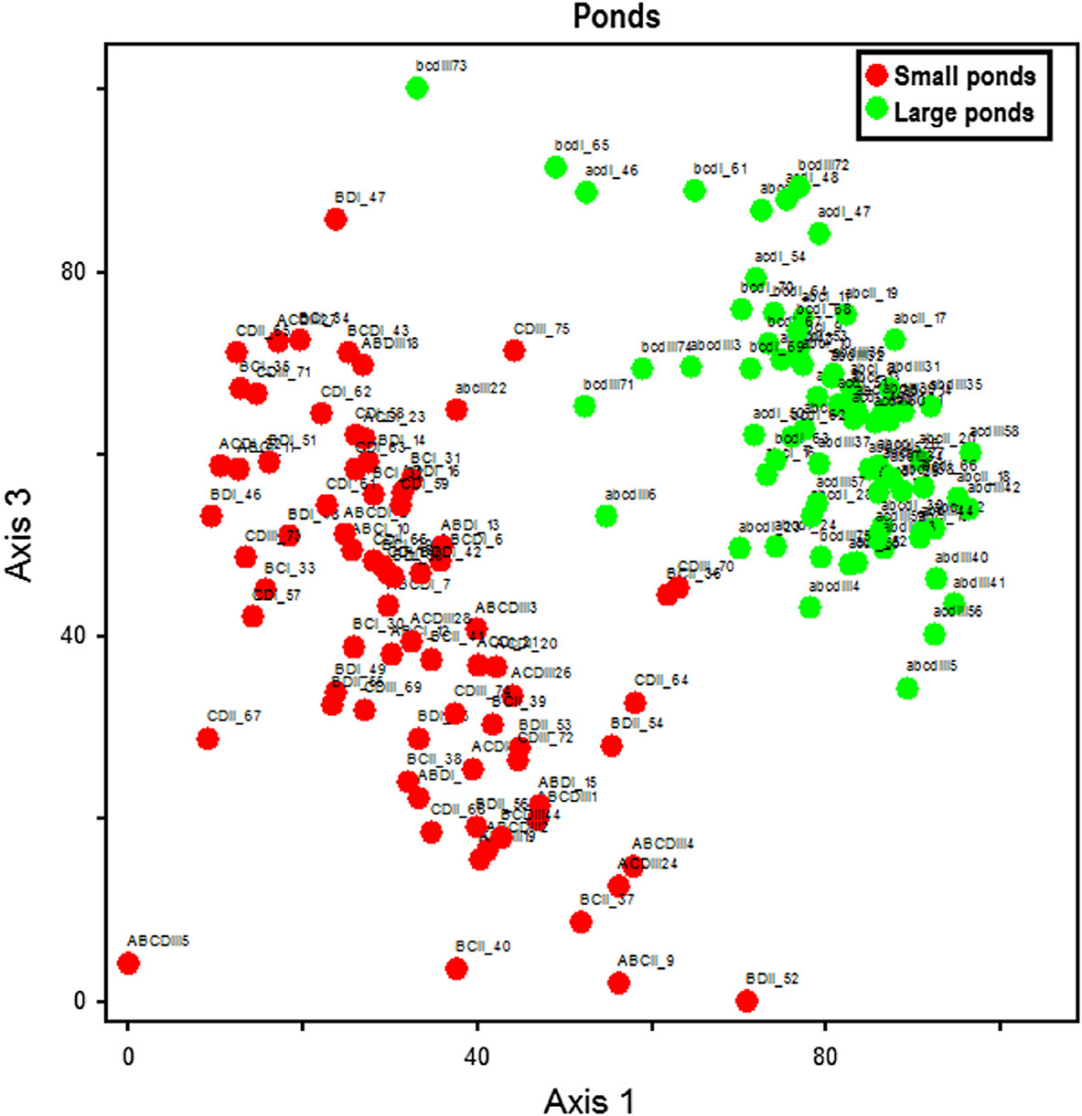
**NMS ordination of ponds based on species structure in freshwater artificial ponds containing tadpoles.** 1: small ponds; 2: large ponds A: *Hoplobatrachus occipitalis*; B: *Kassina fusca*; C: *Ptychadena bibroni*; D: *Phrynomantis microps*

In temporary artificial ponds, the algal species structure also remained an important biological variable. Indeed, the NMS ordination of ponds based on the composition of recorded microalgae species in both small and large artificial ponds indicates that the composition of microalgae differed in relation to the size of the ponds. The structure and species composition significantly varied between small ponds and large ponds (Figure 4). Furthermore, the relative abundance of algal groups in artificial freshwater ponds was correlated with some measured physicochemical variables (Table 4). At p < 5%, chlorophyte biomass was positively correlated with water depth (r = 0.37; p = 0.000), transparency (r = 0.20; p = 0.015) and the pH of water (r = 0.22; p = 0.006). Water depth was also positively correlated with the biomass of euglenophytes (r = 0.17; p = 0.034) and negatively correlated with the biomass of diatoms (r = -0.17; p = 0.042). Dissolved oxygen was negatively correlated only to the biomass of cyanoprocaryotes (r = -0.18; p = 0.029). Nitrates, ammonium (NH_4_^+^) and the phosphate-phosphorus concentration were negatively correlated with the biomass of chlorophytes (r = -0.16 to -0.19; p = 0.044-0.017), while water temperature was negatively correlated with the biomass of chlorophytes (r = -0.25; p = 0.002) but positively correlated with diatom richness (r = 0.21; p = 0.008).

**Table 4:** **Correlation between algal richness in each community and environmental variables in artificial ponds**

		**pH**	**EC**	**Transp.**	**Depth**	**O**_**2**_	**NO**_**3**_^**−**^	**PO**_**4-P**_	**NH**_**4**_^**+**^	**T**
Chlorophytes	r	.225	-.132	.197	.376	-.015	-.165	-.168	-.195	-.251
	p	.006*	.107	.015*	.000*	.858	.044*	.040*	.017*	.002*
Euglenophytes	r	.034	.105	.101	.173	-.018	-.111	-.091	.018	-.149
	p	.677	.200	.218	.034*	.827	.175	.268	.830	.069
Diatoms	r	-.061	.022	-.013	-.166	-.141	-.128	-.002	.120	.215
	p	.459	.790	.872	.042	.085	.119	.983	.144	.008*
Dinophytes	r	.086	.038	.033	-.041	.082	.041	-.076	-.066	-.113
	p	.294	.645	.692	.620	.315	.620	.353	.420	.169
Cyanoprocaryotes	r	.091	-.114	.025	-.149	-.179	-.143	.001	-.077	.148
	p	.269	.164	.760	.069	.029*	.081	.996	.348	.071

*: significant correlation at p < 0.05. EC: Electrical conductivity; Transp.: Transparency.

**Figure 5:**
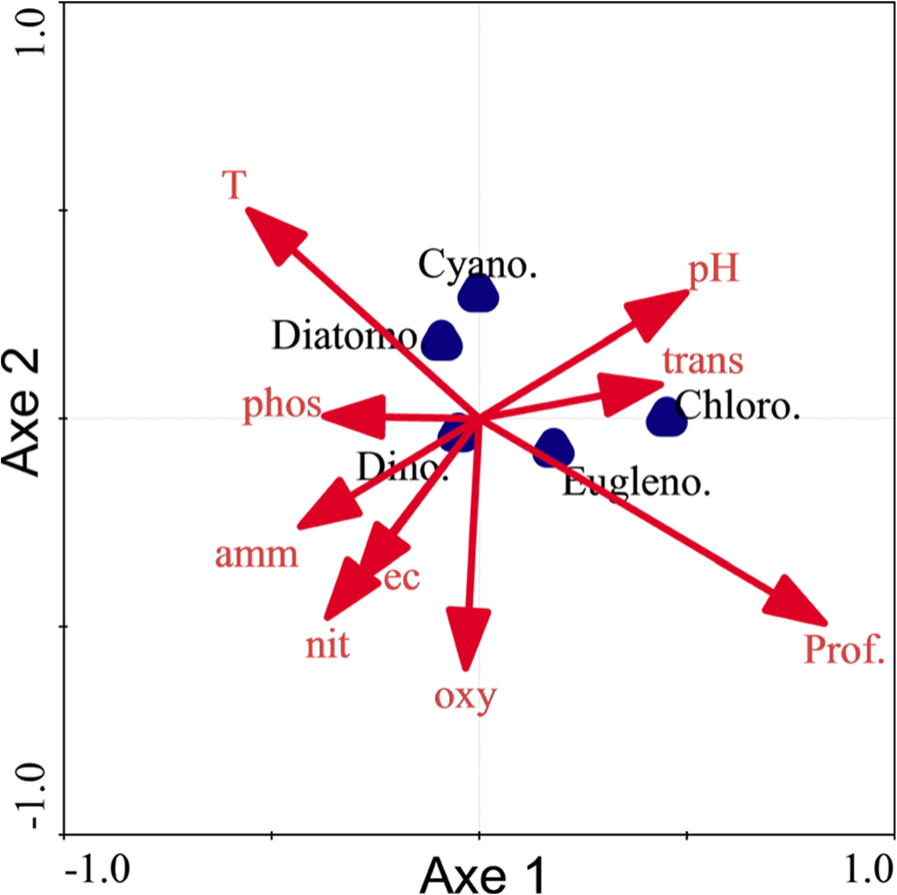
**RDA indicating position of algal groups and vectors of environmental variables.** Chloro: Chlorophytes, Cyano: Cyanophytes, Diatomo: Diatomophyceae (Diatoms), Dino: Dinoflagellates (Dinophytes), Eugleno: Euglenophytes; ec: Electrical conductivity, Dep: depth, transp: transparency, nit: nitrates, Phos: phosphates-phosphorus, amm: ammonium, oxy: dissolved oxygen.

The first four axes of the redundancy analysis (RDA) of algal communities and environmental variables accounted for 100% of the total variance in artificial freshwater ponds (Figure 5). The correlation between the algal richness of communities and environmental variables along the first axis was 45% and 30% along the second axis. Each of the two axes explained 20.5% of the variance of the composition and relative abundance of the algal groups. The first axis was dominated by water depth and transparency (r = 0.37). The second axis was dominated by water depth, which was nevertheless weakly correlated to this axis (r = 0.08). Different correlations between algal communities and physicochemical variables were also revealed by the redundancy analysis (Figure 5). During the analysis, the Monte Carlo permutation test showing the degree of correlation between variables and the richness of communities indicated that algal richness in temporary artificial ponds was deeply and significantly affected by transparency (F = 24; p = 0.002), depth (F = 24; p = 0.002) and the nitrate concentration in water (F = 7; p = 0.012).

### Microalgae and tadpole assemblages

**Figure 6:**
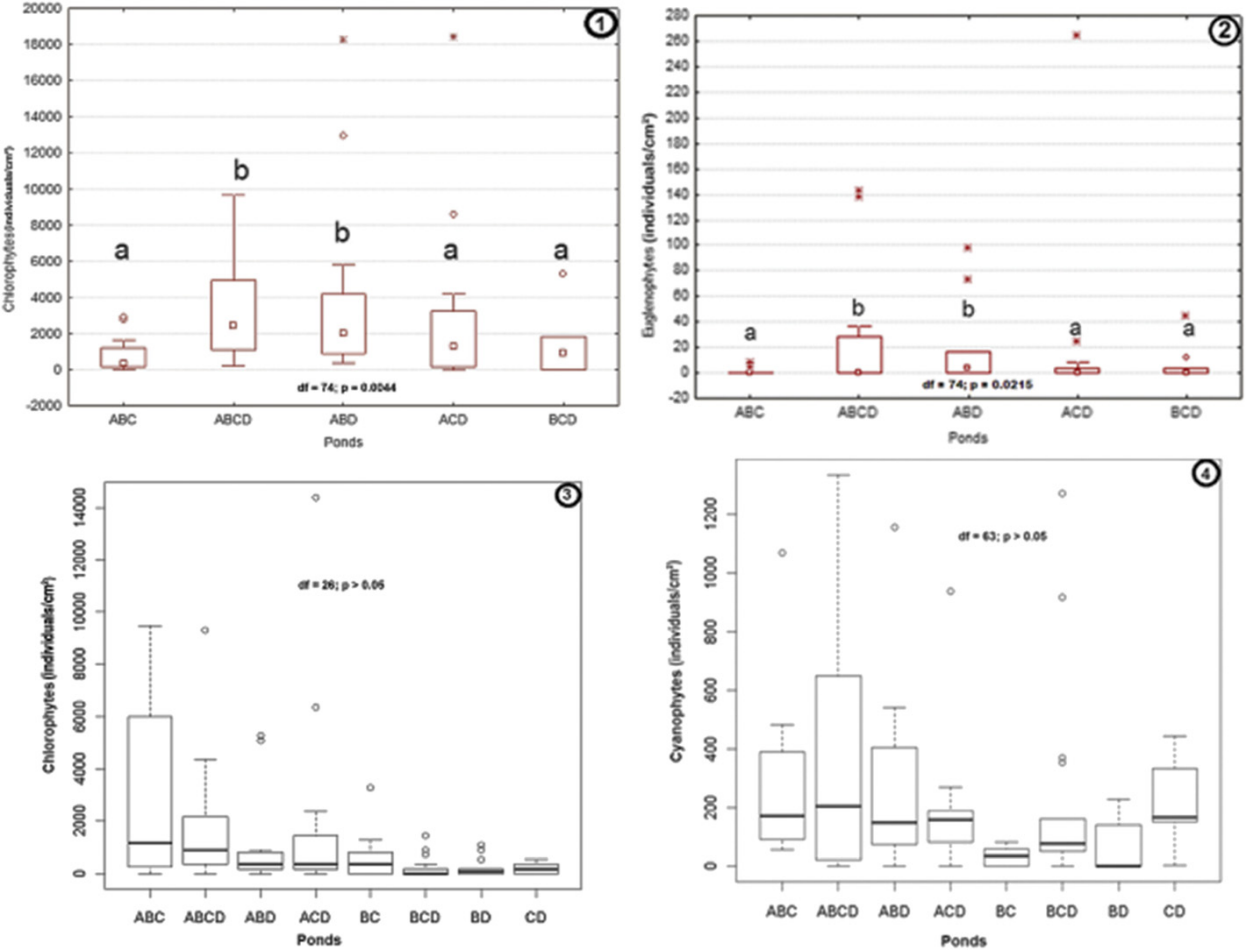
**Comparison of community biomass between tadpole assemblages using a t-test. 1**: abundance of chlorophytes from periphyton in large ponds; **2**: abundance of euglenophytes from periphyton in large ponds; **3**: abundance of chlorophytes within periphyton in small ponds; **4**: abundance of cyanophytes within periphyton in small ponds. A: *Hoplobatrachus occipitalis*; B: *Kassina fusca*; C: *Ptychadena bibroni*; D: *Phrynomantis microps*. Small letters indicate significant differences between treatments.

The biomass of the algal communities was varied in function for periphyton and phytoplankton (Table 5). A comparison of the community biomass in periphyton between tadpole assemblages using the Wilcoxon test coupled with a t-test showed significant differences (e.g., Figure 6 (1-2)). In small ponds, differences in dinoflagellates (F = 4; p = 0.0011) and diatoms were observed (F = 3; p = 0.0136). The biomass of dinoflagellates was higher in ponds with BCD than in ponds with ABCD, ABC, ABD, ACD, BC, or CD. The biomass of diatoms was lower in ponds with ABC ACD, BCD, and ACD than in ponds with BD. In large ponds, differences in biomass were observed in chlorophytes (F = 4; p = 0.0137), cyanophytes (F = 3; p = 0.0238) and euglenophytes (F = 5; p = 0.0215). The biomass of chlorophytes was lower in ponds containing ABC and BCD than in ponds containing ABCD, ACD, and ABD, while the biomass of cyanophytes was higher in ponds containing ABCD, ACD, and BCD than in ponds with ABC or ABD. Euglenophyte biomass was lower in ponds with ABC than in ponds containing ABD.

**Table 5:** **p-values of algal community biomass variation in artificial freshwater ponds**

	**Small ponds**		**Large ponds**	
**Taxonomic groups**	**Periphyton**	**Phytoplankton**	**Periphyton**	**Phytoplankton**
Chlorophytes	p > 0.05	p = 0.045	p = 0.013	p = 0.004
Euglenophytes	p > 0.05	p > 0.05	p = 0.021	p > 0.05
Diatoms	p = 0.013	p > 0.05	p > 0.05	p > 0.05
Dinophytes	p = 0.001	p = 0.038	p > 0.05	p > 0.05
Cyanoprocaryotes	p > 0.05	p > 0.05	p = 0.023	p = 0.020

Variation is significant at p < 0.05.

The comparison of community biomass in phytoplankton showed differences only for dinoflagellates in small ponds (F = 2; p = 0.0386). For this group, the abundance of algae was higher in ponds with BCD than in ponds with ABC or ACD. In large ponds, differences were observed in the abundance of cyanophytes (F = 4; p = 0.0201) and chlorophytes (F = 2; p = 0.0044). The abundance of cyanophytes in ponds containing ABC, ABD, ACD, and BCD was lower compared to ponds containing ABCD. The abundance of chlorophytes in ponds containing ABCD or ABD was higher than ponds containing ABC, ACD, and BCD.

However, insignificant differences were also observed in some artificial ponds at p < 0.05 (Figure 6 (3,4)). Thus, we note in Figure 6 (3) that the abundance of chlorophytes from periphyton was relatively higher in large ponds containing ABC and ABCD than in ponds containing ABD, ACD, BC, BCD, BD, and CD. Chlorophyte abundance was also relatively higher in ponds with ABD and ACD than in ponds with BC, BCD, BD, and CD. The same remark is observed in Figure 6 (4) with cyanophyte abundance in small artificial ponds.

## Discussion

### Effects of physicochemical variables on algae

Microalgae have short generation times, and they react rapidly to changes in the environment. In freshwater temporary ponds, algal richness and abundance were higher at the end (14 days after) of the experiment compared to at the start. Algal biomass increased remarkably in artificial temporary ponds from the beginning to the end of the experiment, which ran for only 14 days. Changing conditions in some physicochemical parameters, such as pH, conductivity and the salinity gradient, had some influence [4] on the algal species distribution in ponds. Different species have different requirements and tolerance ranges for various environmental factors [20]. Nevertheless, physicochemical variables were important factors for species richness and abundance in communities within artificial ponds. These physical and chemical properties of water [21] are central to the physiology and ecology of freshwater organisms. Among them, water transparency and water depth were important to the survival of microalgae (e.g., chlorophytes, cyanophytes, and diatoms) in freshwater artificial ponds. These factors determine the rate of light penetration, which is essential for the development and growth of algae.

The dominance of chlorophytes in small and large ponds was due to shallow water and a higher light availability in these aquatic habitats. During experimentation, both large and small artificial ponds had low water levels (mean depth < 0.5 m). Water bodies with a low water level and therefore a higher light availability are generally inhabited by chlorophytes and various other life forms but mostly lack cyanophytes. The latter ones develop mainly in more eutrophic, light-limited environments [3]. With a higher light availability, algae generally form large colonies (e.g., *Paulschulzia* sp.) of filamentous or large and rounded unicellular organisms, such as chlorophytes. The morphology of the cells, the cell-aggregates, and their ability to change their dimensions under different environmental conditions, coupled with their physiology, may be of great importance in determining chlorophyte settling and floating velocities and thereby act on their capabilities in light harvesting and nutrient exploitation [3].

Nutrients such as nitrogen and phosphorus are found within freshwater habitats in a wide range of forms and are essential to all living organism [21]. In freshwater temporary ponds, the negative correlation of algal biomass of communities with nitrates, ammonium and phosphate-phosphorus concentrations may be explained by the presence of some tadpoles, which reduce algal biomass. The nutrient level in artificial ponds was very low. The nutrients essentially came from the soil that was placed in the experimental tanks, and the rainwater used to fill these containers was poor in nutrients. With low concentrations in ponds, nitrates, ammonium and phosphates-phosphorus could also be limiting factors of algal development. The observed algal biomass was lower than in natural ponds because of the lower levels of nutrients. Yet, lake biota, such as phytoplankton and bacteria, have the ability to survive in low nutrient level environments [21]. Low concentrations of nutrients, particularly phosphates and nitrates, indicate the absence of eutrophication and a high water quality in artificial ponds. That signifies the absence of human impact (pollution from urban and agricultural sources) on freshwater artificial ponds unlike natural ponds. However, a high concentration of nutrients in natural aquatic systems results in low water quality and the excessive growth of phytoplankton and cyanoprocaryotes [6]. Algae show a wide variation, with different species being adapted to acid, neutral and alkaline waters. In freshwater artificial ponds, the pH was neutral, with mean values between 7-7.3. All of the species reported in ponds can be considered to be neutrophiles according to Sigee [21], who considers neutrophiles as species growing in a range pH of 5.5-8.5.

Diatoms are a particular group of algae in aquatic ecosystems. They inhabit the widest variety of aquatic ecosystems [22] and have a high sensitivity to environmental variables. Thus, diatoms are used to characterize water quality in ecosystems (e.g., [23-25]). However, diatoms were only strongly correlated with water transparency in small ponds. The absence of a strong correlation with other water parameters in this group could be explained by the weak variation of these parameters in the artificial ponds during the experiments. Moreover, silicate, an important component for the growth of diatoms [22], was not measured during our experiments; a low silicate level in the artificial ponds might have induced the lower presence of diatoms.

### Effects of tadpole assemblages on algae

The differences in algal richness and abundance between tadpole functional groups in artificial ponds indicate that tadpoles can influence the structure and dynamic of algae in tropical ponds [13,15]. However, the effects of tadpoles on algae can vary [15] from direct (e.g., grazing) to indirect (e.g., the effect of tadpoles on water quality, which regulates the community structure of microalgae) [9,26]. Tadpoles (e.g., *Kassina fusca*, *Ptychadena bibronii*, *Phrynomantis microps*) that are herbivorous, detritivorous and filter-feeders, respectively, have an impact on the algal structure in freshwater habitats [10,11]. Grazer presence [15,27] and total tadpole feeding reduce algal biomass [28,29]. In natural ecosystems, freshwater snails are also found to feed on and reduce primary production [30]. In freshwater artificial ponds, the composition and species structure as well as abundance for the periphyton community were more affected by tadpoles than for phytoplankton. Indeed, the periphyton community was dominated by chlorophytes essentially composed of filamentous algae (e.g., *Mougeotia* sp., *Oedogonium* sp.), which were more vulnerable to the effects of grazers and detritivores. Waringer-Löschenkohl & Schagerl [31] found that *Rana dalmatina* larvae grazed on filamentous algae (*Oscillatoria*, *Spirogyra*, *Chladophora*) as well as pelagic algae (*Scenedesmus*, *Chlamydomonas*, *Cryptomonas*). The grazing activity primarily serves to produce a suspension [32]. Nevertheless, detritivorous tadpoles of *P. bibroni* were often seen to graze the walls of the tanks where a large number of periphyton grew as well. This would reduce their effect on periphyton grown on blades because grazers feed where the periphyton is more abundant. According to Hillebrand and Kahlert [27], biotic interactions, such as grazing and nutrient regulation, have been found to be important for the colonization of hard substrata by microalgae in freshwater habitats. However, tadpoles can remove suspended particles, including phytoplankton, from the water column [33,29] because all free-living tadpoles, except for some specialized microphagous forms, can suspension-feed [32].

In the phytoplankton community, chlorophytes were the group showing a higher significant difference between tadpole assemblages because of the presence of the filamentous algae and large unicellular organisms in this group. However, phytoplankton was dominated by colonies of cyanophytes (e.g., *Microcystis aeruginosa*, *Chroococcus limneticus*) and Chlorophytes (e.g., *Paulschulzia* sp.), with few filamentous algae. The absence of the influence of tadpoles on some communities of phytoplankton (e.g., euglenophytes, cyanophytes in small ponds; euglenophytes, dinophytes, diatoms in large ponds) could have two explanations. First, unlike periphyton, phytoplankton are pelagic (not fixed to any support) and thus more difficult to be directly affected by tadpoles besides filter-feeding tadpoles and to some extent the filter-feeding activities of *Kassina fusca* and even *Hoplobatrachus occipitalis*. Second, the phytoplankton was dominated by colonies of unicellular algae, which have smaller sizes among microalgae. Nevertheless, tadpole grazing apparently reduced the availability of suspended particles, including phytoplankton [34,29]. They were not appreciably affected by herbivorous and detritivorous tadpoles. The significant responses of phytoplankton communities observed in ponds could result from filter-feeding by *Phrynomantis microps* tadpoles. Then, the difference in the responses of algal communities to tadpole influences could reside in the difference in morphology of microalgae in ponds because microalgae have a wide range of vegetative morphologies [35]. Tadpoles can be specialized for microphagy or macrophagy. Microphagous tadpoles are specialized for ingesting the earliest primary productivity, which develops in temporary ponds, namely, small phytoplankton, while microphagous larvae graze on macrophytes and periphyton [32].

The difference in the microalgal community structure between assemblages due to the presence or absence of the predaceous tadpole *H. occipitalis* was probably a result of the different survival rates of amphibian larvae. Tadpoles have low survival rates when *H. occipitalis* tadpoles (A) are present [36]. Predation by *H. occipitalis* tadpoles considerably decreases the numbers of co-occurring tadpole species in ponds. The predation effect decreases or even eliminates the possible impact of tadpoles in ponds. In this study, the predatory tadpoles of *H. occipitalis* influenced the survival rates of *K. fusca*, *P. microps* and *P. bibroni*. Significantly, more larvae survived when the predator tadpoles were absent [37]. Grazing could be an important factor of differences noticed in microalgal communities in ponds. This factor seemed to have a high impact in some ponds where predators were absent (Figure 6 (3,4)), and the abundance of chlorophytes and cyanophytes, for example, seemed to be low with different assemblages of *P. microps*, *P. bibroni* and *K. fusca*. With the presence of carnivorous tadpoles of *H. occipitalis* in assemblages, the abundance was higher. Hence, the community assemblage of tadpoles was important for determining the structure and composition of algae in freshwater temporary ponds.

However, the presence of *Phrynomantis microps* tadpoles in ponds could decrease the impact of predators and favour the impact of other tadpoles. By ingesting particles, they can increase water clarity. Increasing the water clarity might negatively influence the hunting success of predators, and tadpoles should thereby profit from such changes in predation risk [38]. Clarity is a major determinant of the condition and productivity [39] of an ecosystem. A change in water transparency in ponds may positively influence the composition structure and abundance of algae in such ponds. Yet, this tendency of increasing algal biomass was not necessarily observed because high biomass due to water transparency was reduced at the same time by herbivores or detritivores. This shows the importance of tadpoles in the regulation of algal communities in natural ponds. Tadpole exclusion experiments are a valuable method for investigating the effect of tadpoles on algal structure in aquatic ecosystems [12]. The effect becomes stronger with increasing tadpole density. In this study, the difference in the community structure of microalgae between small and large artificial ponds (Figure 4) could likely be due to the difference in tadpole density between the two types of ponds (high density in small ponds and low density in large ponds). Where abundant, tadpoles significantly reduced algal growth and they altered the algal community composition [9]. A severe reduction of tadpoles will thus significantly increase algal biomass, alter the algal assemblage structure and increase the accumulation of organic and inorganic sediments on the substratum [16]. Other animals, such as mosquito larvae and zooplankton, that feed on algae are competing with tadpoles for food [28,30]. They are also the prey of *Hoplobatrachus*, and they disturb the algal structure. Each species is an important element of the food web, contributing to the balance of temporary ponds. A reduction in the amphibian population would contribute to the impoverishment of ecosystems that could negatively influence aquatic and terrestrial lives.

## Conclusion

In freshwater artificial ponds, the algal community was sensitive to physicochemical variables and tadpole assemblages. The environmental variables of ponds determined the structure of the algal community in these ecosystems. Transparency and pond size (particularly the depth of ponds) seemed to be the physicochemical factors that most significantly affected the algal community structure. Low concentrations of nutrients indicated higher water quality and lower abundances of microalgae in artificial ponds compared to natural ponds exposed to human activities where lower water quality and higher abundances of algae are observed, which are signs of eutrophication [40].

Differences in the algal community structure between some treatments could be the effect of the different influence of tadpole assemblages on this structure. The variation in the community of tadpoles influences food web dynamics and energy flow [16] in their habitats. Various responses of algal communities to the different assemblages of tadpoles in artificial freshwater ponds could be observed. Herbivorous tadpoles of *K. fusca*, detritivorous tadpoles of *P. bibroni* and filter-feeder tadpoles of *P. microps* affect algal community structure in freshwater temporary ponds. A decreasing population density of these species may negatively affect the water quality of their habitats and thereby negatively affect aquatic and terrestrial ecosystems.

## Methods

### Study area

**Figure 7:**
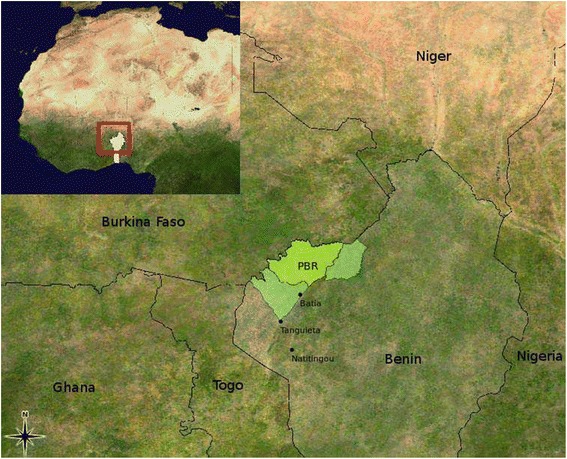
**Study area.**

Investigations were conducted in the Pendjari area (10° 30′-11° 30′ N and 0° 50′-2° 00′ E), a reserve of flora and fauna with approximately 5000 km^2^ located in the extreme northwest of Benin in West Africa (Figure 7). The experiments were conducted in Batia, a village located at the southern edge of the reserve. This area is characterized by the Sudanian climate, with a rainy season (May-October) and a dry season (November-April). The mean annual temperature is 27°C, and the mean annual precipitation is approximately 1000 mm. The vegetation is a mixture of different savannah types with mostly open shrub and tree savannah. Small forest islands composed of dry forest patches and open gallery forests along watercourses are present. Most savannah waters are temporary and form only after heavy rains in May/June and dry up in October/November [41]. In this area, temporary ponds are particularly numerous and used for breeding by many amphibian species. The amphibian species richness of the Pendjari area is among the most diverse of African savannahs. The species assemblage is mainly composed of typical West African savannah frogs, especially those restricted to drier habitats [41].

### Experimental procedures

**Figure 8:**
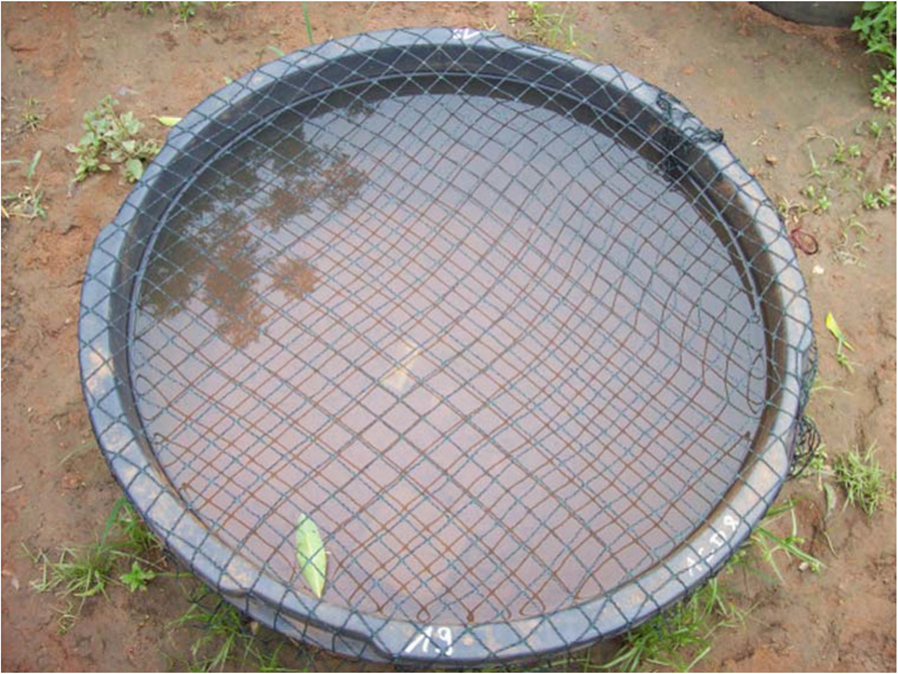
**Photograph of small artificial temporary ponds (volume: 90 L).**

**Figure 9:**
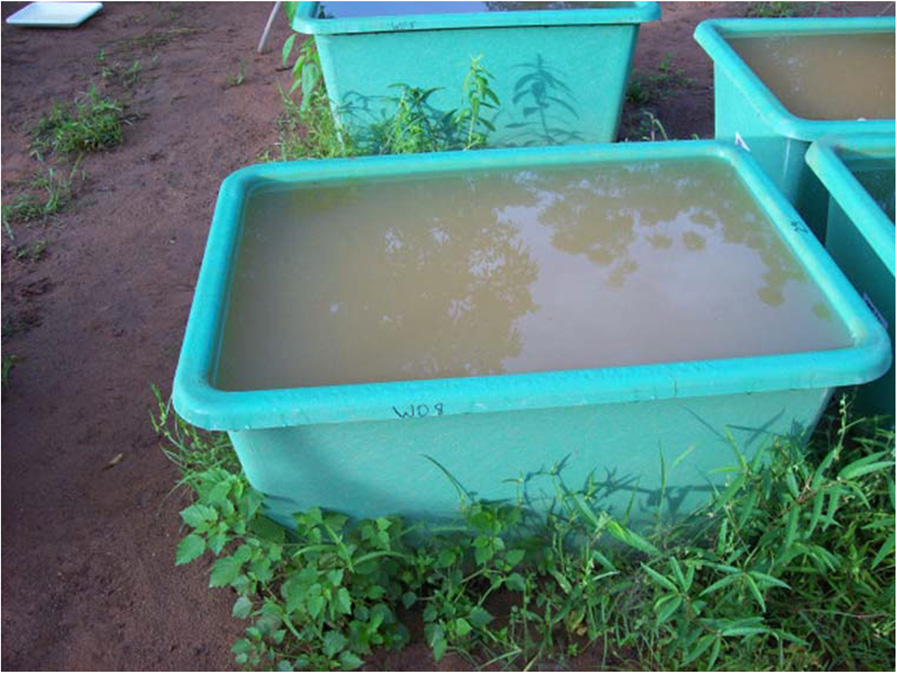
**Photograph of large artificial temporary ponds (volume: 200 L).**

In total, 78 tanks were used to conduct the experimental work during the rainy seasons of 2007 and 2008. Two types of tanks (Figures 8 and 9) were used as artificial temporary ponds: forty (40) small tanks with a volume of 90 L (small artificial ponds) and thirty eight (38) large tanks with a volume of 200 L (large artificial ponds). To set up artificial freshwater ponds, the soil of a dried up natural pond was used as the sediment at the bottom of the tanks. Afterwards, the tanks were filled with rainwater. Before tadpole assemblages entered the tanks, we collected samples of phytoplankton and placed four microscopic slides on two different plastic supports on the bottom of each tank for the collection of periphyton. Tadpoles of four different species (*Hoplobatrachus occipitalis* (A), *Kassina fusca* (B), *Ptychadena bibroni* (C), *Phrynomantis microps* (D)) were then placed into the ponds in different combinations (ABCD, ABC, ABD, ACD, BCD, BC, BD, CD). Each species belongs to a different trophic level.

Tadpoles of *H. occipitalis* are very effective predators, which are able to rapidly reduce the number of other tadpoles [42,43]. They generally inhabit shallow water without vegetation and the pond ground. Tadpoles of *K. fusca* are herbivorous. Younger stages prefer vegetation-rich areas in shallower water. The larvae of *P. bibroni* are mainly detritivores, while the larvae of *P. microps* filter-feed in the upper water column. Table 6 shows the density of each species introduced in the small and large artificial ponds.

**Table 6:** **Distribution of tadpoles and their assemblages in small and large artificial ponds**

**Species**	**Diet**	**Small ponds**	**Large ponds**
*Hoplobatrachus occipitalis* (A)	Carnivorous	1	1
*Kassina fusca* (B)	Herbivorous	20	20
*Ptychadena bibroni* (C)	Detritivorous	10	20
*Phrynomantis microps* (D)	Filter-feeding	20	20
*ABCD*	-	51	61
*ABC*	-	31	41
*ABD*	-	41	41
*ACD*	-	31	41
*BCD*	-	50	60
*BC*	-	30	40
*BD*	-	40	40
*CD*	-	30	40

The experiments were conducted from July to September. Each experimental run was conducted over a period of 14 days. All experiments were performed at the same time during the first and second years (2007 and 2008). During the experiment periods, each tadpole assemblage was replicated at least five times in different small tanks but also in different large tanks. However, experiments were performed in conjunction with the availability of the tadpole species. *K. fusca* tadpoles were only available at the end of August and beginning of September.

Depth, transparency, conductivity, and pH were measured daily. Nitrate (NO_3_^−^), ammonium (NH_4_^+^) and phosphate-phosphorus (PO_4_-P) concentrations were measured at the start and end of each experiment. Samples of phytoplankton and periphyton were also collected, and the number of tadpoles of each species was counted at the end of the experiments. During the experiments, small tanks were specially protected by wire netting in 2007 and 2008 because as small tanks had a lower height than large tanks, adult amphibians were able to easily enter these small tanks and disturb the experiment. Additionally, in 2008, a fence surround of the artificial ponds was set up to prevent other animals, especially amphibians, from entering the tanks (a problem encountered in 2007). In a few cases, adult amphibians entered the tanks for oviposition. Those artificial ponds were excluded from the experiment, and a further experiment was performed. Nevertheless, during each phase of the experiments, the ponds were totally exposed to natural environmental factors and their changes.

### Algae sampling procedures

Samples of phytoplankton were collected using a pipette with a volume of 120 ml. They were taken from ponds at the beginning and end of each experiment. All samples were preserved with a 5% solution of formaldehyde.

For periphyton, microscopic slides were placed at the bottom of ponds to allow the growth of species of periphyton to look at the effects of tadpoles on this group. Slides on which periphyton developed were removed at the end of the experiment and immediately transferred to bottles already containing pond water enriched with 5% of formaldehyde for preservation.

### Measurements of physicochemical parameters

Measurements of water temperature, pH, electrical conductivity, and salinity were performed with Hanna® Instruments Combo-Tested HI 98129. Transparency was measured with a Secchi disk, and the depth was taken with a graduate band. The concentration of nitrates, phosphates-phosphorus and ammonium were measured using the colorimetric tests of Machery-Nagel Visocolor Eco (range of measurements: NH_4_^+^: 0 - 15 mg/l; NO^3−^: 0 - 120 mg/l; PO_4_-P: 0 - 5 mg/l).

### Analysis of algal samples

In the laboratory, samples of microalgae were prepared and observed with a photonic microscope (Olympus) to identify species and determine their abundance in samples. Sub-samples of 50 ml were taken from phytoplankton samples and then kept for 24 h for the sedimentation of organisms according to Bourrelly [44]. Then, the upper water was removed with a micropipette, and the rest was used for observation with the photonic microscope to spot the different species inside each sample. The different species reported in a sample collected from a specific tank were considered as species richness of that tank at the time of collection (start or end of experiment). To determine the relative abundance of phytoplankton in each pond, a Fuchs-Rosenthal chamber was used [45]. The chamber was filled with a homogeneous sample, and the filled chamber was left undisturbed for 5 minutes to allow particles to settle [46]. After settling, all of the individuals in the chamber were counted. A repetitive counting of individuals in the chamber was performed four times for each sample, and the mean abundance of species was calculated.

Periphyton on microscopic slides was directly observed in the microscope to determine the species and their abundance. Regarding species richness, the whole surface of each blade with periphyton was analysed, and the different species were recorded. On four randomly selected points of 3.1 mm^2^, the blade individuals per species were counted, and the mean values were taken on one square centimetre calculated.

### Classification of species in systematic groups

All of the recorded species of algae in the different ponds were classified in the following taxonomic groups: cyanobacteria (Cyanophytes: blue-green algae), chlorophytes (green algae), euglenoid algae (euglenophytes), bacillariophytes (diatoms), and dinophytes (dinoflagellates). This classification was based on a combination of characteristics, including photosynthetic pigments, starch reserve products, cell covering and other aspects of cellular organization [47].

### Data analysis

Non-metric multidimensional scaling (NMS) was used to describe variation among microalgal community structure between ponds. Using NMS ordination allows the assessment of whether the community assembly of microalgae followed similar trajectories among environmental variables in artificial ponds. Ordination analyses were performed using the presence/absence of species in each artificial temporary pond. We used NMS because it is highly suitable for ecological data containing numerous zero values [48]. The lowest stress value was used as the starting coordinates for analysis. NMS ordination allowed for the separation of the different treatments during experiments and was performed using the program PC-ORD version 4 [49].

However, to distinguish the levels of influence of different environmental variables on microalgal communities, CANOCO Program Version 4.5 [50] was used. In this program, the species richness of communities (taxonomical groups) was first analysed using detrented component analysis (DCA) to measure the gradient lengths of different axes. The gradient length allowed for the selection of the type of analyses (unimodal analyses or linear analyses) to run the data. If the length is lower than 4 SD, unimodal analysis is recommended, but if it is higher than 4 SD, then linear analysis is recommended. Because the results of the DCA showed that the gradient lengths (maximum: 1.54) were lower than 4 SD, we used a linear method (redundancy analysis: RDA) to analyse our data.

Regression analyses were also used to measure the correlation between communities and different environmental variables. These analyses allowed us to examine the possible correlations between algal community richness and variables in ponds and whether an environmental variable was able to influence the community structure of microalgae in ponds.

To discern differences in microalgal communities between tadpole assemblages, a Wilcoxon comparison coupled with a t-test was also used. This analysis permitted us to find communities that differed significantly in biomass between certain tadpole assemblages. Thus, we considered groups obtained by taxonomic classification of different species in ponds as communities of microalgae in ponds. The Wilcoxon test, t-test and regression tests were performed using the statistical software “R” and “Statistica 7.1”.

To execute the different statistical analyses above, data collected at the end of each experiment were used.

## Abbreviations

NMS: Non-metric multidimensional scaling
RDA: Redundancy analysis
DCA: Detrented component analysis
SD: Standard deviation
